# Should Preoperative Biliary Decontamination Be Considered to Minimize Morbidity and Mortality Following Pancreatoduodenectomy?

**DOI:** 10.3390/antibiotics15020134

**Published:** 2026-01-29

**Authors:** Natalia Olszewska, Tomasz Guzel, Agnieszka Milner, Piotr Paluszkiewicz, Edyta Podsiadły, Maciej Słodkowski

**Affiliations:** 1 Department of General Gastroenterological and Oncological Surgery, Medical University of Warsaw, 02-097 Warsaw, Poland; nattalia.olszewska@gmail.com (N.O.); piotr_paluszkiewicz@o2.pl (P.P.); 2 Microbiological Laboratory, University Center for Laboratory Medicine, Medical University of Warsaw, 02-097 Warsaw, Poland; agnieszka.milner@uckwum.pl; 3 Department of Dental Microbiology, Medical University of Warsaw, 02-097 Warsaw, Poland; edyta.podsiadly@uckwum.pl

**Keywords:** antibiotics, bacteriobilia, pancreatoduodenectomy, pancreatic cancer, antimicrobial stewardship, postoperative complications, postoperative pancreatic fistula

## Abstract

Background: Pancreatoduodenectomy (PD) remains the fundamental treatment for periampullary malignancies but is associated with considerable morbidity (20–50%) and mortality (2–7%). Bacteriobilia contributes to unfavourable postoperative outcomes. Current antibiotic prophylaxis recommendations endorse first-generation cephalosporins, which often fail to adequately target pathogens most frequently isolated from bile. To date, no specific guidelines for preoperative targeted antibiotic therapy have been established, although tailoring such strategies to the bile microbiome may improve surgical outcomes. This study aimed to characterize bile microbiology in patients undergoing PD for pancreatic ductal adenocarcinoma (PDAC), evaluating potential antibiotherapy regimens that provide effective coverage against the most frequently isolated pathogens. Methods: A retrospective cohort analysis of 725 patients surgically treated for pancreatic tumours at a high-volume pancreatic surgery center between 2017 and 2022 was performed. To minimize heterogeneity, study was restricted to 138 patients who underwent PD with histopathological confirmed PDAC. Intraoperative bile cultures were assessed. Results: Patients with bacteriobilia likewise experienced worse outcomes: higher 5-year mortality (OR 3.01, *p* = 0.007), greater overall postoperative pancreatic fistula (POPF) occurrence (OR 2.54, *p* = 0.044) and wound infections (OR 2.90, *p* = 0.038). Among bile microbiome the highest susceptibility rates were observed for combination of amoxicillin/clavulanic acid with gentamicin, while the lowest were noted for cephalosporin–metronidazole regimen (93.6% vs. 30.2%, respectively). Conclusions: Bacteriobilia contributes to postoperative complications and serves as a predictor of poorer survival after PD. Standard perioperative antibiotic prophylaxis in PD is insufficient. Based on our findings, perioperative antibiotic therapy with amoxicillin/clavulanic acid and gentamicin combination appears to provide superior coverage and may improve postoperative morbidity and overall survival following PD.

## 1. Introduction

Pancreatoduodenectomy (PD) remains one of the most technically demanding procedures in gastrointestinal surgery, requiring specialized expertise and multidisciplinary perioperative care. Despite advances in surgical technique and perioperative management, outcomes have remained poor over recent decades: perioperative mortality rates in high-volume centers are reported at 2–7%, and overall morbidity still ranges 20–50% [1,2,3,4,5,6]. Infectious complications dominate this burden. Surgical site infections (SSI) occur in up to one-third of patients, and clinically relevant postoperative pancreatic fistula (POPF) in 7–33%, with severe POPF as a leading cause of sepsis, prolonged hospitalization, and delay in adjuvant chemotherapy [3,5,6,7,8,9,10].

Most patients with periampullary malignances present with obstructive jaundice. Preoperative biliary drainage (PBD), commonly achieved by endoscopic retrograde cholangiopancreatography (ERCP) with stent placement, is frequently required but comes at a cost: biliary colonization rates reach 90% after PBD compared with far lower rates in non-drained patients [5,7,11,12,13]. Typical pathogens isolated from intraoperative bile cultures include *Enterococcus* spp., *Klebsiella pneumoniae*, *Enterobacter cloacae*, and *Escherichia coli*, with a substantial proportion displaying resistance to standard perioperative prophylactic regimens [3,6,14]. Colonized bile has been consistently linked to higher postoperative infectious morbidity, and emerging evidence suggests that pathogenic organisms may also worsen survival in patients with pancreatic ductal adenocarcinoma (PDAC) [3,5,6].

Yet, current international perioperative antibiotic prophylaxis guidelines are not tailored to the microbiological reality of PD. The World Health Organization’s (WHO) recommendations for SSI prevention, as well as the American Society of Health-System Pharmacists/Infectious Diseases Society of America/Surgical Infection Society/Society for Healthcare Epidemiology of America (ASHP/IDSA/SIS/SHEA), and European societies guidelines still classify PD as “clean-contaminated” gastroduodenal surgery, recommending first-generation cephalosporins in prophylaxis, such as cefazolin [9,10,11]. Numerous studies demonstrate that this regimen does not match the microbiological profile of infected bile or postoperative SSI [3,4,5,7,14]. Hence, perioperative antibiotic treatment is not a standard procedure before PD. In an era of routine PBD and increasing antimicrobial resistance, the mismatch between recommended prophylaxis and actual pathogens is striking.

These observations underscore the need to re-evaluate antibiotic strategies in PD. Tailoring perioperative antibiotherapy to local bile microbiology may reduce postoperative infection, shorten hospital stay, and ultimately improve both short- and long-term outcomes.

The present study therefore aims to characterize the bacterial colonization patterns of bile in patients undergoing PD for PDAC at our institution and to identify antibiotic regimens that provide optimal coverage for the most frequently isolated organisms.

## 2. Material and Methods

### 2.1. Study Design and Patients Selection

A retrospective analysis was performed of 725 patients admitted for surgical treatment of pancreatic tumors at the Department of General, Gastroenterological, and Oncological Surgery between January 2017 and December 2022. The clinic is recognized as high volume pancreatic center [15]. To ensure a homogeneous and methodologically robust study population, inclusion was restricted to patients who underwent PD with a histopathological diagnosis of PDAC. Based on these criteria, 138 patients were eligible for inclusion.

### 2.2. Surgical Technique

Pancreatoduodenectomies were performed by three experienced pancreatic surgeons using the artery-first approach. Depending on tumor location and intraoperative assessment, either the classical Whipple procedure or the pylorus-preserving Traverso–Longmire technique was applied. Pancreatic reconstruction was achieved by pancreato-enteric anastomosis, using either duct-to-mucosa or invagination technique, while biliary continuity was restored with hepaticojejunostomy tailored to duct diameter and condition. Gastrointestinal continuity was re-established through gastrojejunostomy with Braun anastomosis or duodenojejunostomy, according to intraoperative findings.

Standard oncological lymphadenectomy was performed using the Heidelberg technique to ensure complete regional nodal clearance [16]. Perioperative antibiotic prophylaxis was initiated 30 min prior to skin incision and consisted of 2 g cefazolin combined with 500 mg metronidazole, with intraoperative re-dosing performed at 4 h intervals for cefazolin and 8 h intervals for metronidazole when indicated.

The study received approval from the Bioethics Committee of the Medical University of Warsaw. All procedures were conducted in accordance with the ethical standards and with the principles outlined in the Declaration of Helsinki. Each patient provided written informed consent for treatment, in accordance with institutional and ethical guidelines.

### 2.3. Microbiological Assessment

Intraoperative bile samples were collected immediately following transection of the common bile duct, using a sterile scalpel blade and swab under strictly aseptic conditions. Specimens were subsequently cultured on standard bacteriological media in accordance with laboratory protocols. Microbial identification was carried out by matrix-assisted laser desorption/ionization time-of-flight (MALDI-TOF) mass spectrometry using the Microflex LT system (Bruker, Germany) and MBT Compass IVD software (Bruker Daltonics, Bremen, Germany), following the manufacturer’s instructions. Antimicrobial susceptibility testing was performed using the Kirby–Bauer disk diffusion method, with interpretation based on the European Committee on Antimicrobial Susceptibility Testing (EUCAST) recommendations. Bacterial strains harboring specific resistance mechanisms (BRM) were defined as drug-resistant organisms capable of surviving antimicrobial exposure through specialized enzymatic or structural adaptations. Carbapenemase production (including metallo-β-lactamases (MBL), Klebsiella pneumoniae carbapenemase (KPC), and OXA-48 was assessed using the double-disk synergy test with EDTA (DDST-EDTA) for MBL, the combined disk test (CDT) for KPC, and temocillin-containing disks for OXA-48 detection. In addition, Rapidec Carba NP and/or the GeneXpert real-time PCR assay (Cepheid, Sunnyvale, CA, USA) were employed for confirmation. Extended-spectrum β-lactamase (ESBL) production was identified using phenotypic confirmatory testing with the double-disk synergy test (DDST). All microbiological data, including bile cultures, were retrieved from the institutional microbiology database. Due to technical limitations related to server migration and data loss, antibiogram results were unavailable for 14 patients. Consequently, the final analysis of antimicrobial resistance patterns was performed in 124 patients.

### 2.4. Statistical Analysis

All statistical analyses were conducted using Statistica 14.0.0. (TIBCO Software Inc., Palo Alto, CA, USA) and IBM SPSS Statistics version 27.0 (IBM Corp., Armonk, NY, USA). Categorical variables are expressed as frequencies and percentages. Comparisons between groups were performed using the chi-square or Fisher’s exact test for categorical data. Odds ratios (ORs) with corresponding 95% confidence intervals (CIs) were calculated to assess the strength of associations between risk factors and outcomes. A two-sided *p*-value < 0.05 was considered statistically significant.

In this study, a retrospective analysis of bacterial susceptibility to antibiotics was performed using a custom-built database comprising three main groups of clinical isolates: Gram-positive cocci, Gram-negative rods, and anaerobic bacteria. Data were extracted from laboratory reports and manually compiled into structured Excel spreadsheets. For each bacterial strain, susceptibility to a panel of antibiotics was assessed and classified into one of the following categories: “S” (Susceptible), “SIE” (Susceptible, Increased Exposure), or “R” (Resistant). The analysis focused on calculating the proportion of susceptible isolates relative to the total number of strains evaluated for each antibiotic. Subsequently, combination therapies involving two antibiotics were virtually simulated to assess potential synergy—assuming that an isolate is treatable if it is susceptible to at least one of the agents in the pair. Top-performing combinations were selected based on the highest cumulative coverage across all isolates. All data processing, statistical summaries, and visualizations were performed using Python 3.13 (pandas was used for data processing and summarization, while matplotlib was applied to visualize antibiotic effectiveness and comparative plots) and Microsoft Excel.

## 3. Results

The distribution of postoperative complications after PD is presented in Table 1. In the subgroup analysis, prior ERCP was associated with a markedly higher risk of adverse outcomes, including increased 5-year mortality (OR 5.57, 95% CI 2.48–12.54; *p* = 0.001) and a higher rate of SSI (OR 4.98, 95% CI 1.63–15.15; *p* = 0.002), while correlating with a lower incidence of bile leak (OR 0.27, 95% CI 0.084–0.868; *p* = 0.021). Patients with positive intraoperative bile cultures likewise experienced worse outcomes: higher 5-year mortality (OR 3.01, 95% CI 1.33–6.80; *p* = 0.007), greater overall POPF and POPF grade B occurrence (OR 2.54, 95% CI 1.01–6.38; *p* = 0.044; OR 5.11 (0.648–40.29), *p* = 0.046), and more SSIs (OR 2.90, 95% CI 1.03–8.15; *p* = 0.038), together with a lower risk of bile leak (OR 0.21, 95% CI 0.066–0.694; *p* = 0.006). Isolation of bacteria with resistance mechanisms (BRM) identified a particularly high-risk subgroup, with excess in-hospital mortality (OR 4.83, 95% CI 1.55–45.84; *p* = 0.004), an increased need for reoperation (OR 4.17, 95% CI 1.24–14.02; *p* = 0.014), and a greater incidence of severe POPF (Grade C) (OR 4.97, 95% CI 1.07–23.07; *p* = 0.026). No other comparisons in Table 1 reached statistical significance.

Subgroup analysis of microorganisms isolated from intraoperative bile samples show distinct and clinically relevant risk profiles (Table 2).

Isolation of Gram-positive cocci was associated with higher 5-year mortality (OR 2.92, 95% CI 1.396–6.10; *p* = 0.004) and an increased rate of SSI (OR 2.36, 95% CI 1.109–5.038; *p* = 0.024), and demonstrated a borderline association with POPF Grade B (OR 3.10, 95% CI 0.947–10.15; *p* = 0.052. Presence of Gram-negative rods likewise correlated with excess 5-year mortality (OR 2.52, 95% CI 1.193–5.323; *p* = 0.014) but, interestingly, with a lower risk of bile leak (OR 0.63, 95% CI 0.077–0.825; *p* = 0.016). In contrast, anaerobic isolates were linked to POPF Grade B (OR 4.63, 95% CI 1.458–14.69; *p* = 0.005), clinically relevant POPF (OR 2.99, 95% CI 1.050–8.523; *p* = 0.034), and SSI (OR 2.69, 95% CI 1.023–7.061; *p* = 0.040). No outcome reached statistical significance in the fungal subgroup. Collectively, these findings indicate that the biliary microbial signature is associated with distinct postoperative risk profiles: mortality correlates with Gram-positive and Gram-negative flora, whereas the risk of POPF and SSI increases significantly in the presence of anaerobes and Gram-positive cocci.

**Figure 1:**
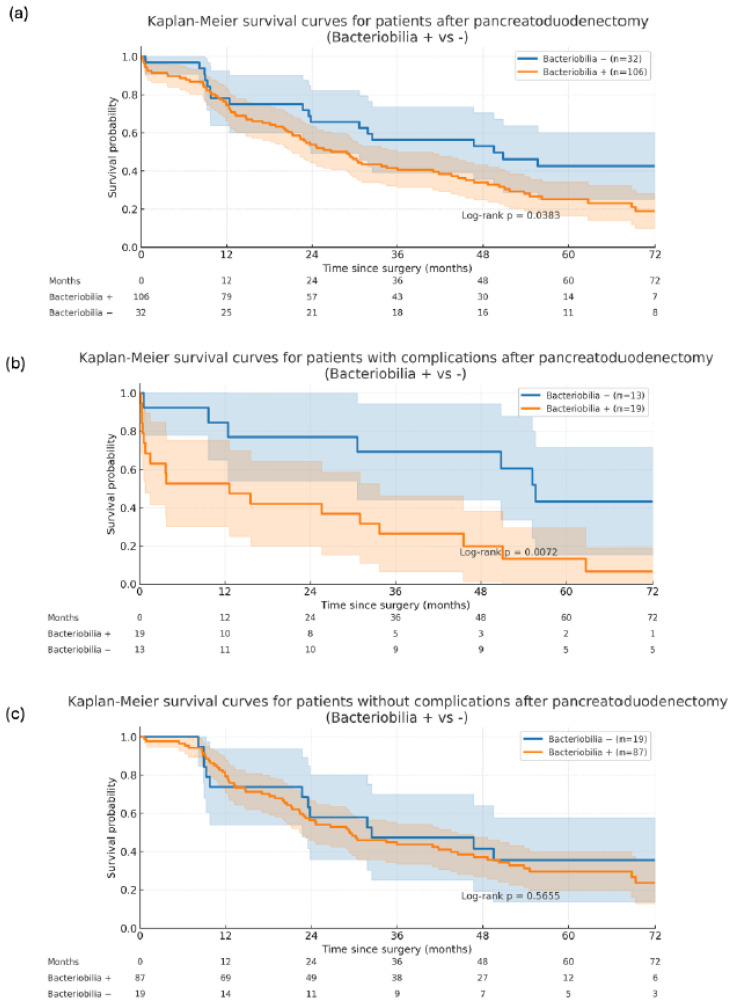
Kaplan–Meier survival curves for patients with and without bacteriobilia (**a**) in subgroups with (**b**) and without (**c**) postoperative complications after pancreatoduodenectomy.

Kaplan–Meier survival analysis demonstrated consistently worse overall survival in patients with bacteriobilia compared with those without (log rank test, *p* = 0.038), and regardless of the occurrence or not of postoperative complications. In the subgroup without or with minor complications (Clavien–Dindo group 1–2), this difference did not reach statistical significance (log rank test, *p* = 0.566). However, among patients who developed severe postoperative complications (Clavien–Dindo 3–5), the presence of bacteriobilia was associated with a significantly poorer OS (log rank test, *p* = 0.007) (Figure 1).

Bile contamination was predominantly polymicrobial. Among the 124 patients analyzed, a total of 252 microbial isolates were identified, including 235 isolates of bacteria (144 Gram-negative rods, 72 Gram-positive cocci, 19 anaerobes (61.3%, 30.6%, 8.1%, respectively)), and 16 fungi. BRM was identified in 23 isolates of bacteria (9.8%) (Table 3).

**Figure 2:**
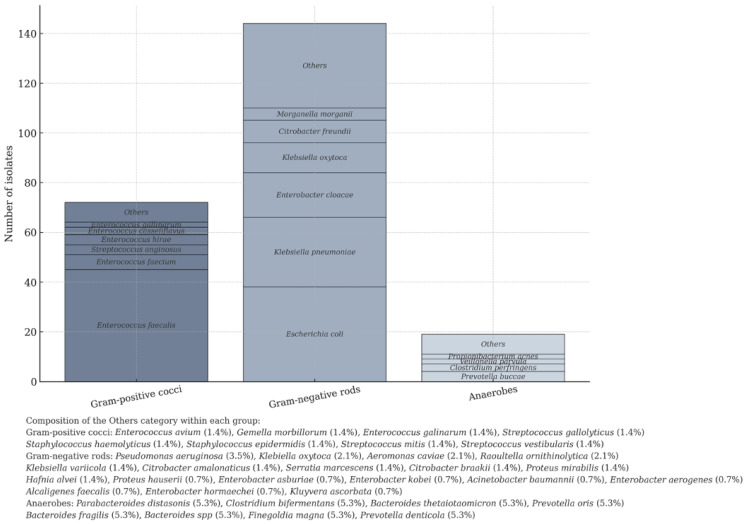
Characteristic of bacterial isolates in bile.

The distribution of bacteria species is illustrated in Figure 2.

Bile colonized by microorganisms was subjected to antimicrobial susceptibility testing in order to characterize resistance patterns and to identify the most effective antibiotic agents providing the broadest spectrum of coverage against the isolated strains. The analysis revealed the highest susceptibility rates to piperacillin–tazobactam, followed by meropenem, gentamicin, amoxicillin–clavulanic acid, and amikacin. Conversely, cefuroxime, a second-generation cephalosporin, exhibited the lowest in vitro activity, even at elevated testing concentrations. Notably, susceptibility data for cefazolin—a first-generation cephalosporin widely employed in perioperative prophylaxis—were unavailable, as this antibiotic is not routinely incorporated into standard susceptibility testing protocols, as it is used just for perioperative antibiotic prophylaxis to prevent surgical site infection caused by residual skin bacterial flora (Table 4).

To further optimize empirical antimicrobial coverage, we analyzed susceptibility profiles to identify combinations of two antibiotics that would provide the broadest spectrum of activity against the isolated strains obtained from intraoperatively collected bile. The most effective combinations were Amoxicillin–Clavulanic acid with Gentamicin, or with Meropenem, covering 93.6% and 92.8% of isolates, respectively. Interestingly, to approximate the effectiveness of commonly used perioperative prophylaxis—based on cefazolin and metronidazole—we performed an additional analysis using another cephalosporin, cefuroxime, in combination with metronidazole. This regimen demonstrated poor coverage, achieving activity against only 30.2% of isolates (Table 5).

## 4. Discussion

Our results suggest that standard perioperative antibiotic prophylaxis currently recommended for pancreatoduodenectomy provides insufficient coverage against the microorganisms most frequently isolated from bile. Using cefuroxime as a surrogate for guideline-recommended cephalosporins, we observed susceptibility in only 23% of isolates, even at escalated doses. By contrast, the combination of amoxicillin/clavulanic acid with gentamicin achieved susceptibility in over 93.6% of isolates, suggesting that broader perioperative antibiotic therapy is required in this setting.

The clinical relevance of biliary colonization is underscored by its high prevalence and impact on outcomes. In our cohort, positive intraoperative bile cultures were identified in 76.8% of patients, and 93.1% in prior ERCP group compared to 32.4% in no prior ERCP group. Those findings are in line with previous reports showing colonization rates exceeding 85–90% in patients with preoperative biliary drainage [14,17,18]. Furthermore, our results show that prior ERCP was associated with markedly worse outcomes, including increased long-term mortality and SSI, while positive bile cultures correlated with higher mortality, greater rates of POPF, and more frequent SSIs.

Microorganism-specific analyses provided further insights. Collectively, our data indicate that mortality is predominantly driven by Gram-positive and Gram-negative flora, whereas fistula formation and wound infections are more strongly associated with anaerobes and Gram-positive organisms. Patients with BRM represented an especially high-risk subgroup, with significantly greater in-hospital mortality, higher rates of reoperation, and severe POPF. Therefore, comprehensive perioperative antibiotic coverage addressing all major bacterial groups is essential to mitigate postoperative risk. Moreover, similar as in Tortajada et al. multicenter research [19], in our study fungal colonization shows no statistically significant association with adverse outcomes. Furthermore, our Kaplan–Meier survival analysis demonstrated that bacteriobilia is an independent risk factor for reduced overall survival after PD, regardless of the occurrence of postoperative complications. These findings suggest that the presence of bacteria in bile should therefore be recognized as an unfavorable prognostic factor associated with poorer survival outcomes after PD.

Notably, current international guidelines emphasize general principles of surgical antimicrobial prophylaxis. The WHO Global Guidelines for the Prevention of Surgical Site Infection (2016) recommend administration of a single dose of first generation cephalosporin within 60 min before skin incision and advise against prolongation beyond the immediate perioperative period in most procedures [9]. Similarly, the joint American guidelines from ASHP, IDSA, SIS, and SHEA stress timely administration, weight-based dosing, intraoperative redosing in long or complex surgeries [10]. Conventional perioperative prophylaxis is intended to prevent superficial SSIs in non-infected patients; it is not tailored to, nor effective for, preventing broader postoperative complications. However, neither WHO nor American surgical guidelines provide specific recommendations for pancreatic surgery, and they do not address the challenge of biliary colonization and resistant organisms. Our results therefore highlight a critical gap between current broad guidance and the specific microbiological realities of PD.

Although European recommendations are more precise. The ECDC technical report on perioperative antibiotic prophylaxis recommends cefazolin as first-line prophylaxis for the Whipple procedure; however, ceftriaxone 2 g or amoxicillin/clavulanic acid 1.2 g may be considered as alternatives. In patients with severe penicillin allergy, it recommends clindamycin 600–900 mg or vancomycin 15–20 mg/kg with gentamicin 5 mg/kg or aztreonam 2 g. The guidance also underscores correct timing, dosing, and discouraging postoperative continuation in most cases [20]. Consistent with our approach, the ERAS Society guidelines for PD acknowledge the high risk of contamination during biliary reconstruction and recommend tailoring antibiotic prophylaxis to the local microbial spectrum, particularly in patients with preoperative biliary drainage (PBD) [21]. A recent systematic review by Steccanella et al. highlighted the heterogeneity of European practice and concluded that cephalosporin-based prophylaxis is often inadequate in hepato-biliary-pancreatic surgeries [11]. Moreover, the ongoing European SPARROW trial is evaluating pre-emptive and extended antibiotic regimens in high-risk patients, and its central hypothesis mirrors ours—namely, that pre-emptive antibiotic therapy may reduce SSIs following PD [22]. Together, these studies reflect increasing recognition that PD patients represent a unique subgroup in which standard prophylaxis may be insufficient—supporting the rationale for broader, microbiology-driven perioperative antibiotic strategies such as the regimen we propose.

Our results align with the growing literature supporting the use of broader-spectrum agents for perioperative prophylaxis. In a multicenter randomized controlled trial, D’Angelica et al. demonstrated that piperacillin–tazobactam (3.375 or 4.5 g iv administered within 60 min before incision, with intraoperative redosing every 2–4 h until wound closure) was superior to cefoxitin, yielding significant reductions in SSIs, postoperative sepsis, and clinically relevant POPF [23]. The meta-analysis by Kumar et al., along with additional independent trials, supports these observations [24,25,26,27]. These findings underscore the importance of appropriately selected perioperative antimicrobial prophylaxis in improving postoperative outcomes. In our cohort, the cumulative susceptibility of bile isolates to piperacillin–tazobactam was 79.1%, supporting its potential as an empiric prophylactic option in settings with a comparable biliary microbiological profile.

A critical issue related to the extended use of perioperative antibiotics is the risk of promoting antimicrobial resistance (AMR). Inappropriate and excessive antibiotic use has been identified as a major factor contributing to the global rise in AMR, thereby reducing the clinical effectiveness of these agents. However, it must be emphasized that a substantial proportion of PD patients require postoperative therapeutic antibiotics due to infectious complications, with reported rates of postoperative infections ranging from 20% to 40% [2,17,23,25,28,29]. Many of these cases are treated with second- or third-line agents such as piperacillin–tazobactam or carbapenems. Studies have shown that use of extended or postoperative therapeutic antibiotics, particularly in high-risk patients with contaminated bile, reduce infectious complications [13,30,31]. Whereas, in accordance with WHO AWaRe principles, piperacillin–tazobactam is categorized by the WHO as a “Watch” agent, emphasizing the necessity of stewardship to prevent inappropriate utilization and to preserve its therapeutic efficacy. In contrast, both agents in our regimen fall within the “Access” category, justifying first-line selection in PD [32]. Thus, we believe that an effective perioperative regimen may decrease the need for escalation antibiotic therapy in postsurgical period, potentially reducing both morbidity and antimicrobial resistance pressures.

Our findings regarding the superior in vitro efficacy of amoxicillin/clavulanic acid combined with gentamicin are supported by clinical evidence. A recently published prospective study of 63 patients undergoing surgery for periampullary malignancies demonstrated that perioperative prophylaxis with amoxicillin/clavulanic acid (1 g/200 mg) and gentamicin (240 mg), tailored according to intraoperative previously obtained bile samples, significantly reduced infectious complications. The most commonly isolated organisms in that cohort were similar as ours—*Enterococcus* spp. and *Klebsiella* spp., both reliably covered by this regimen [33]. Multiple studies analyzing the bile microbiome have brought concordant results, consistently identifying *Escherichia coli*, *Enterococcus faecalis*, and *Klebsiella pneumoniae* as the predominant isolates [3,6,14,18,27,34,35]. This reproducibility across different cohorts and centers indicates that the bile microbiome demonstrates a relatively stable and predictable composition, irrespective of local variability. Such findings underscore the clinical relevance of considering the bile microbiome in perioperative management, as they provide a strong rationale for establishing standardized empiric antibiotic therapy protocols. Developing universal guidelines tailored to these commonly encountered pathogens may help to optimize perioperative antimicrobial strategies.

Our study has several limitations. As a retrospective analysis from a single center, the results of our study should be interpreted as hypothesis-generating. Retrospective studies play an important role in identifying clinically relevant patterns and potential risk factors, thereby providing a rationale for the design of prospective interventional trials. In this context, our analyses represent the first step in a structured research pathway aimed at addressing this clinically relevant question. The use of cefuroxime as a surrogate for cefazolin in our analysis may not fully reflect standard practice. Nevertheless, our data are consistent with growing evidence that standard cephalosporin-based prophylaxis is inadequate for PD. Given the polymicrobial nature of biliary colonization and the presence of resistant phenotypes, regimen choice should be based on stewardship-driven review and timely de-escalation as culture data become available. Perioperative antibiotic treatment with amoxicillin/clavulanic acid plus gentamicin demonstrated the highest in vitro susceptibility across biliary isolates (93.6%), supporting its candidacy as a regimen with the most favorable antimicrobial profile, with potential to reduce morbidity and improve both short- and long-term outcomes. A prospective randomized trial assessing the proposed antibiotic protocols is ongoing and will further clarify their clinical utility.

## 5. Conclusions

Our findings indicate that bacteriobilia contributes to early postoperative morbidity, including POPF and SSI, and represents a potential prognostic marker for reduced OS after PD. Standard perioperative antibiotic prophylaxis in PD seems to be insufficient and should no longer be considered adequate. Based on our results, perioperative, targeted antibiotic therapy with the amoxicillin/clavulanic acid and gentamicin combination appears to provide superior coverage, low risk of developing antimicrobial resistance and may improve postoperative morbidity and mortality following PD.

## Figures and Tables

**Table 1:** Distribution of postoperative complications after pancreatoduodenectomy across subgroups—prior endoscopic retrograde cholangiopancreatography (ERCP), positive bile culture, and bacteria with resistance mechanisms (BRM).

Variable	Study Population n/N(%)	Prior ERCP n/N(%), OR (95% CI), *p*	Positive Bile Culture n/N(%), OR (95% CI), *p*	BRM n/N(%), OR (95% CI), *p*
Prevalence in study population	138/138 (100%)	101/138 (73.2%)	106/138 (76.8%) ^a^	17/138 (12.3%)
		-	-	-
Overall 5 years mortality	92/138 (66.7%)	78/101 (77.2%)	77/106 (72.6%)	14/17 (82.4%)
		**OR 5.57 (2.476–12.54), *p* = 0.001**	**OR 3.01 (1.332–6.799), *p* = 0.007**	OR 2.57 (0.700–9.452), *p* = 0.143
In-hospital mortality	6/138 (4.3%)	5/101 (5.0%)	5/106 (4.7%)	3/17 (17.6%)
		OR 1.88 (0.212–16.60), *p* = 0.566	OR 1.54 (0.173–13.64), *p* = 0.699	**OR 8.43 (1.550–45.84), *p* = 0.004**
Reoperation	16/138 (11.6%)	12/101 (11.9%)	12/106 (11.3%)	5/17 (29.4%)
		OR 1.11 (0.335–3.693), *p* = 0.862	OR 0.89 (0.267–2.990), *p* = 0.855	**OR 4.17 (1.238–14.02), *p* = 0.014**
POPF	51/138 (37%)	38/101 (37.6%)	44/106 (41.5%)	6/17 (35.3%)
		OR 1.11 (0.507–2.444), *p* = 0.788	**OR 2.54 (1.007–6.378), *p* = 0.044**	OR 0.92 (0.319–2.661), *p* = 0.879
POPF Grade B	16/138 (11.6%)	14/101 (13.9%)	15/106 (14.2%)	2/17 (11.8%)
		OR 2.82 (0.608–13.04), *p* = 0.169	**OR 5.11 (0.648–40.29), *p* = 0.046**	OR 1.02 (0.211–4.933), *p* = 0.981
POPF Grade C	8/138 (5.8%)	5/101 (5.0%)	5/106 (4.7%)	3/17 (17.6%)
		OR 0.59 (0.134–2.603), *p* = 0.482	OR 0.48 (0.108–2.123), *p* = 0.323	**OR 4.97 (1.071–23.07), *p* = 0.026**
Clinically relevant POPF	24/138 (17.4%)	20/101 (19.8%)	21/106 (19.8%)	5/17 (29.4%)
		OR 1.58 (0.546–4.570), *p* = 0.396	OR 1.73 (0.547–5.469), *p* = 0.347	OR 2.10 (0.667–6.634), *p* = 0.197
Bile leak	13/138 (9.4%)	6/101 (5.9%)	6/106 (5.7%)	2/17 (11.8%)
		**OR 0.27 (0.084–0.868), *p* = 0.021**	**OR 0.21 (0.066–0.694), *p* = 0.006**	OR 1.33 (0.269–6.606), *p* = 0.724
Bleeding	9/138 (6.5%)	6/101 (5.9%)	6/106 (5.7%)	0/17 (0.0%)
		OR 0.72 (0.170–3.022), *p* = 0.648	OR 0.58 (0.137–2.463), *p* = 0.456	OR 0.93 (0.889–0.974), *p* = 0.245
Wound infection (SSI)	42/138 (30.4%)	38/101 (37.6%)	37/106 (34.9%)	6/17 (35.3%)
		**OR 4.98 (1.635–15.15), *p* = 0.002**	**OR 2.90 (1.029–8.147), *p* = 0.038**	OR 1.29 (0.442–3.748), *p* = 0.642
Readmission	17/138 (12.3%)	12/101 (11.9%)	12/106 (11.3%)	2/17 (11.8%)
		OR 0.86 (0.282–2.641), *p* = 0.796	OR 0.69 (0.223–2.129), *p* = 0.516	OR 0.94 (0.196–4.535), *p* = 0.941
ICU admission	12/138 (8.7%)	9/101 (8.9%)	9/106 (8.5%)	3/17 (17.6%)
		OR 1.11 (0.283–4.339), *p* = 0.882	OR 0.90 (0.228–3.533), *p* = 0.876	OR 2.67 (0.645–11.03), *p* = 0.162
Wirsungostomy	4/138 (2.9%)	3/101 (3.0%)	3/106 (2.8%)	1/17 (5.9%)
		OR 1.10 (0.111–10.94), *p* = 0.934	OR 0.90 (0.091–8.992), *p* = 0.931	OR 2.46 (0.241–25.08), *p* = 0.434

^a^ 94/101 (93.1%)—in prior ERCP group; 12/37 (32.4%)—in no prior ERCP group.

**Table 2:** Distribution of postoperative complications after pancreatoduodenectomy across subgroups of microorganisms—Gram-positive cocci, Gram-negative rods, anaerobes and fungi.

Variable	Gram-Positive Cocci n/N(%), OR (95% CI), *p*	Gram-Negative Rods n/N(%), OR (95% CI), *p*	Anaerobes n/N(%), OR (95% CI), *p*	Fungi n/N(%), OR (95% CI), *p*
Prevalence in study population	72/138 (52.2%)	94/138 (68.1%)	19/138 (13.8%)	16/138 (11.6%)
		-	-	-
Overall 5 years mortality	56/72 (77.8%)	69/94 (73.4%)	17/19 (89.5%)	13/16 (81.2%)
	**OR 2.92 (1.396–6.10), *p* = 0.004**	**OR 2.52 (1.193–5.323), *p* = 0.014**	OR 3.25 (0.900–11.72), *p* = 0.060	OR 2.36 (0.637–8.734), *p* = 0.188
In-hospital mortality	4/72 (5.6%)	5/94 (5.3%)	1/19 (5.3%)	0/16 (0.0%)
	OR 1.88 (0.333–10.63), *p* = 0.467	OR 2.42 (0.274–21.32), *p* = 0.413	OR 1.19 (0.132–10.75), *p* = 0.877	OR 0.95 (0.913–0.990), *p* = 0.364
Reoperation	9/72 (12.5%)	10/94 (10.6%)	1/19 (5.3%)	0/16 (0.0%)
	OR 1.20 (0.422–3.440), *p* = 0.728	OR 0.75 (0.255–2.225), *p* = 0.608	OR 0.36 (0.045–2.900), *p* = 0.319	OR 0.87 (0.811–0.931), *p* = 0.123
POPF	31/72 (43.1%)	37/94 (39.4%)	9/19 (47.4%)	4/16 (25.0%)
	OR 1.74 (0.862–3.510), *p* = 0.121	OR 1.39 (0.652–2.967), *p* = 0.392	OR 1.48 (0.568–3.860), *p* = 0.420	OR 0.53 (0.162–1.746), *p* = 0.292
POPF Grade B	12/72 (16.7%)	12/94 (12.8%)	6/19 (31.6%)	1/16 (6.2%)
	OR 3.10 (0.947–10.15), *p* = 0.052	OR 1.46 (0.444–4.825), *p* = 0.530	**OR 4.63 (1.458–14.69), *p* = 0.005**	OR 0.48 (0.059–3.865), *p* = 0.478
POPF Grade C	4/72 (5.6%)	4/94 (4.3%)	1/19 (5.3%)	0/16 (0.0%)
	OR 0.91 (0.219–3.802), *p* = 0.899	OR 0.44 (0.106–1.867), *p* = 0.257	OR 0.84 (0.097–7.172), *p* = 0.869	OR 0.93 (0.892–0.978), *p* = 0.291
Clinically relevant POPF	16/72 (22.2%)	17/94 (18.1%)	7/19 (36.8%)	1/16 (6.2%)
	OR 1.81 (0.739–4.433), *p* = 0.191	OR 0.99 (0.392–2.515), *p* = 0.989	**OR 2.99 (1.050–8.523), *p* = 0.034**	OR 0.27 (0.034–2.164), *p* = 0.190
Bile leak	5/72 (6.9%)	5/94 (5.3%)	0/19 (0.0%)	1/16 (6.2%)
	OR 0.54 (0.168–1.746), *p* = 0.298	**OR 0.25 (0.077–0.825), *p* = 0.016**	OR 0.89 (0.835–0.948), *p* = 0.119	OR 0.61 (0.074–5.041), *p* = 0.644
Bleeding	5/72 (6.9%)	4/94 (4.3%)	1/19 (5.3%)	0/16 (0.0%)
	OR 1.16 (0.297–4.504), *p* = 0.834	OR 0.35 (0.088–1.361), *p* = 0.115	OR 0.724 (0.086–6.121), *p* = 0.766	OR 0.93 (0.881–0.974), *p* = 0.261
Wound infection (SSI)	28/72 (38.9%)	31/94 (33.0%)	10/19 (52.6%)	6/16 (37.5%)
	**OR 2.36 (1.109–5.038), *p* = 0.024**	OR 1.48 (0.659–3.307), *p* = 0.342	**OR 2.69 (1.023–7.061), *p* = 0.040**	OR 1.43 (0.485–4.239), *p* = 0.514
Readmission	10/72 (13.9%)	10/94 (10.6%)	1/19 (5.3%)	3/16 (18.8%)
	OR 1.36 (0.486–3.806), *p* = 0.558	OR 0.63 (0.222–1.781), *p* = 0.380	OR 0.34 (0.042–2.682), *p* = 0.281	OR 1.78 (0.451–7.029), *p* = 0.405
ICU admission	7/72 (9.7%)	9/94 (9.6%)	3/19 (15.8%)	0/16 (0.0%)
	OR 1.31 (0.396–4.361), *p* = 0.655	OR 1.45 (0.372–5.631), *p* = 0.592	OR 2.14 (0.525–8.693), *p* = 0.279	OR 0.90 (0.850–0.956), *p* = 0.189
Wirsungostomy	3/72 (4.2%)	2/94 (2.1%)	1/19 (5.3%)	0/16 (0.0%)
	OR 2.83 (0.287–27.86), *p* = 0.354	OR 0.46 (0.062–3.352), *p* = 0.430	OR 2.02 (0.199–20.42), *p* = 0.545	OR 0.97 (0.936–0.999), *p* = 0.462

**Table 3:** Distribution of most commonly presented microbial isolates in bile samples.

Microorganism Group	Species (Examples)	Number of Isolates	% of Total Isolates in Groups
Anaerobes n = 19	*Prevotella buccae*	4	21.1% of Anaerobes
	*Clostridium perfringens*	3	15.8% of Anaerobes
	*Veilionella parvula*	2	10.5% of Anaerobes
	*Bacteroides* spp.	3	15.8% of Anaerobes
Gram-negative rods (GNR) n = 144	*Escherichia coli*	38	26.4% of GNR
	*Klebsiella pneumoniae*	28	19.4% of GNR
	*Enterobacter cloacae*	18	12.5% of GNR
	*Klebsiella oxytoca*	15	10.4% of GNR
	*Citrobacter freundii*	9	6.2% of GNR
Gram-positive cocci (GPC) n = 72	*Enterococcus faecalis*	45	62.5% of GPC
	*Enterococcus faecium*	6	8.3% of GPC
	*Streptococcus anginosus*	4	5.6% of GPC
	*Enterococcus hirae*	4	5.6% of GPC
Fungi n = 16	*Candida albicans*	13	81.2% of Fungi
Bacteria with resistance mechanisms (BRM) n = 23	*Klebsiella pneumoniae ESBL*	7	30.4% of BRM
	*Escherichia coli ESBL*	7	30.4% of BRM
	*Klebsiella pneumoniae ESBL + NMD*	3	13.0% of BRM
	*Enterococcus casseliflavus VRE*	2	8.7% of BRM
	*Enterococcus gallinarum VRE*	1	4.3% of BRM
	*Klebsiella oxytoca ESBL*	1	4.3% of BRM
	*Enterobacter aerogenes ESBL*	1	4.3% of BRM
	*Enterobacter cloacae ESBL*	1	4.3% of BRM

VRE—vancomycin–resistant Enterococcus; ESBL—extended–spectrum beta–lactamases; NDM—New Delhi metallo–β–lactamase.

**Table 4:** Overall resistance (all bacteria combined).

Antibiotic	Sensitive Isolates (n = 235)	Sensitivity (%)	Resistant Isolates (n = 235)	Resistance (%)
Piperacillin/tazobactam	186	79.1	49	20.9
Meropenem	147	62.6	88	37.4
Gentamicin	141	60.0	94	40.0
Amoxicillin/Clavulanic acid	141	60.0	94	40.0
Amikacin	132	56.2	103	43.8
Imipenem	128	54.5	107	45.5
Cefepime	118	50.2	117	49.8
Ciprofloxacin	117	49.8	118	50.2
Trimethoprim/sulfamethoxazole	115	48.9	120	51.1
Aztreonam	105	44.7	130	55.3
Ceftazidime	78	33.2	157	66.8
Ceftriaxone	75	31.9	160	68.1
Tigecycline	69	29.4	166	70.6
Vancomycin	68	28.9	167	71.1
Linezolid	66	28.1	169	71.9
Teikoplanina	64	27.2	171	72.8
Clindamycin	21	8.9	214	91.1
Metronidazole	17	7.2	218	92.8
Cefuroxime ^a^	54	23.0	181	77.0

^a^ Susceptible; Increased Exposure.

**Table 5:** Antibiotic combinations providing the broadest coverage of bile isolates.

Antibiotic 1st	Antibiotic 2nd	Covered Isolates (n = 235)	Coverage (%)
Amoxicillin/Clavulanic acid	Gentamicin	220	93.6
Amoxicillin/Clavulanic acid	Meropenem	218	92.8
Meropenem	Tigecycline	215	91.5
Piperacillin/tazobactam	Gentamicin	215	91.5
Vancomycin	Meropenem	215	91.5
Amoxicillin/Clavulanic acid	Amikacin	214	91.1
Linezolid	Meropenem	213	90.6
Gentamicin	Meropenem	206	87.7
Cefuroxime ^a^	Metronidazole	71	30.2

^a^ Susceptible, Increased Exposure.

## Data Availability

Data is available on request.

## Abbreviations

The following abbreviations are used in this manuscript:

AMR	Antimicrobial Resistance
BRM	Bacteria with Resistance Mechanisms
ERCP	Endoscopic Retrograde Cholangiopancreatography
PBD	Preoperative Biliary Drainage
PC	Pancreatic Cancer
PD	Pancreatoduodenectomy
PDAC	Pancreatic Ductal Adenocarcinoma
POPF	Postoperative Pancreatic Fistula
